# Supplementary material for: IL-20 Cytokines Are Involved in Epithelial Lesions Associated with Virus-Induced COPD Exacerbation in Mice
Source: Biomedicines. 2021 Dec 5;9(12):1838. doi: 10.3390/biomedicines9121838 (PMC8699027; doi:10.3390/biomedicines9121838)
Supplement: Supplementary file 1 [file biomedicines-09-01838-s001.zip › biomedicines-1468369-supplementary.pdf]

IL-20 Cytokines Are Involved in Epithelial Lesions Associated with Virus-Induced COPD Exacerbation in Mice

Mélina Le Roux [1], Anaïs Ollivier [1], Gwenola Kervoaze [1], Thimothé Becke, Laurent Gillet [2], Muriel Pichavant [1] and Philippe Gosset [1,*]

[1] CIIL-Center for Infection and Immunity of Lille, CHRU Lille, Institute Pasteur de Lille, University Lille, CNRS UMR9017, Inserm U1019, 59000 Lille, France; melina.le-roux@pasteur-lille.fr (M.L.R.); anais.ollivier@pasteur-lille.fr (A.O.); gwenola.kervoaze@pasteur-lille.fr (G.K.); timotheebeke@gmail.com (T.B.); muriel.pichavant@pasteur-lille.fr (M.P.)

[2] Immunology-Vaccinology Laboratory, Department of Infection and Parasitic Diseases, FARAH, University of Liege, 4000 Liege, Belgium; L.gillet@ulg.ac.be

* Correspondence: philippe.gosset@pasteur-lille.fr; Tel.: +33-320-877-965

Supplemental Figures

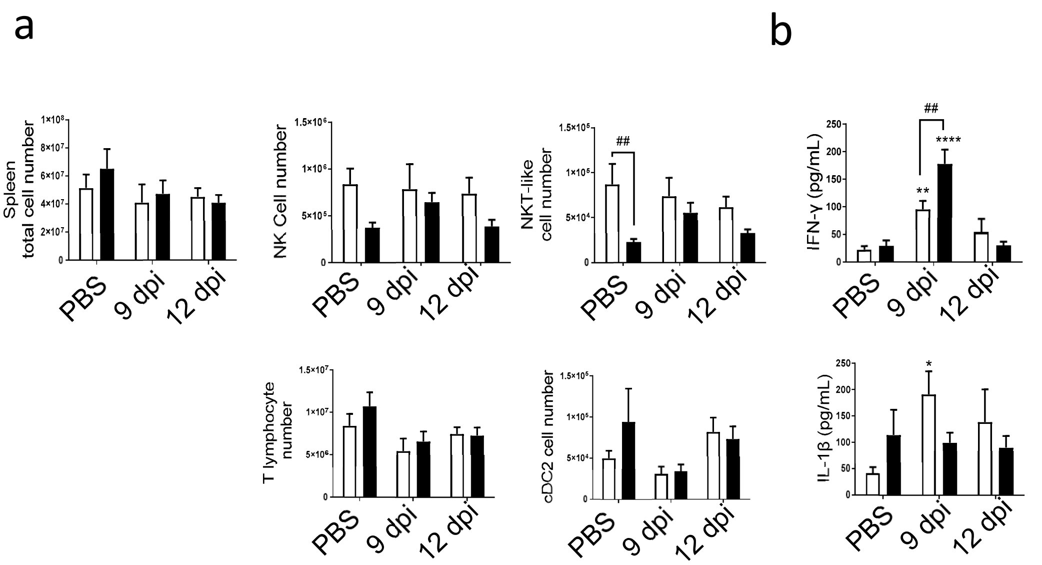

Figure S1. **PVM infection modulates splenic and blood inflammatory response in Air and CS mice.** (**a**) Total cell number, CD45+/TCRβ-/NK1.1+ NK cells, CD45+/TCRβ+/NK1.1+ NKT like cells, CD45+/TCRβ+ T lymphocytes and CD45+/ F4/80-/ CD11c+, CD11b+ dendritic cells (cDC2) count were analysed in spleen tissue of Air (white bars) and CS mice (Balck bars). (**b**) Circulating IFN-γ and IL-1β levels were analysed by ELISA in sera. Data represent the mean ± SEM. * $p < 0.05$, ** $p < 0.01$ and **** $p < 0.0001$ correspond to virus effect (PVM vs PBS). ## $p < 0.01$correspond to CS effect (CS vs. Air)

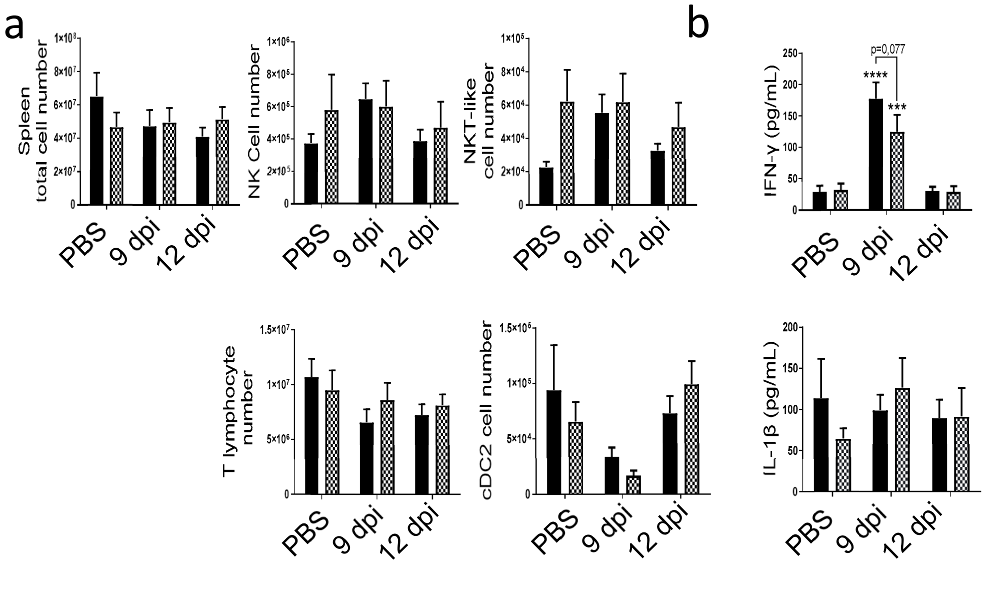

Figure S2. PVM infection modulates splenic and blood inflammatory response in CS-exposed WT and IL-20Rb KO mice. (**a**)Total cell number, CD45+/TCRβ-/NK1.1+ NK cells, CD45+/TCRβ+/NK1.1+ NKT like cells, CD45+/TCRβ+ CD4+ and CD8+ T lymphocytes and CD45+/ F4/80-/ CD11c+/CD11b+ dendritic cells (cDC2) count were analysed in spleen of WT (Black bars) and IL-20Rb KO mice (grey bars). (**b**) Circulating IFN-γ and IL-1β levels were analysed by ELISA. Data represent the mean ± SEM. *** $p < 0.001$ and **** $p < 0.0001$ correspond to virus effect (PVM vs PBS).

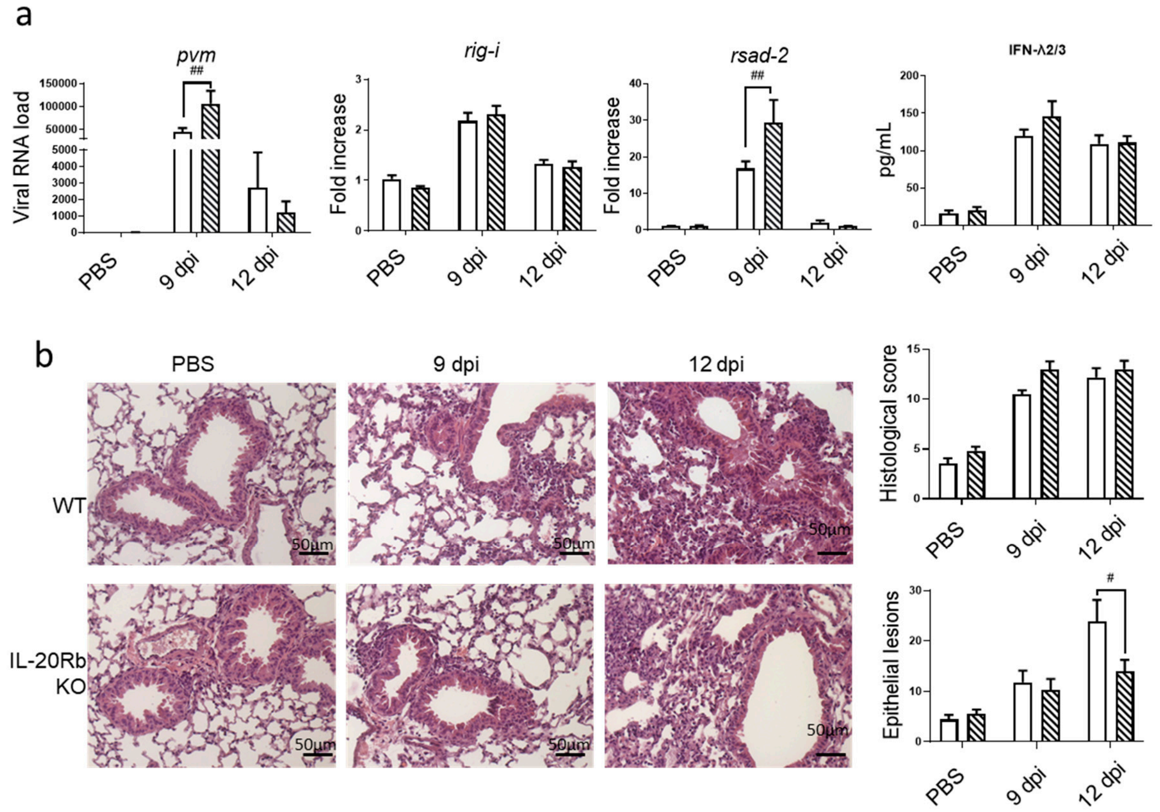

Figure S3. Impact of IL-20Rb depletion on PVM infection in Air-exposed mice. (a) viral load and antiviral response including mRNA exppression *rig-i, rsad-2*, evaluated by RT-qPCR in lung tissues. Results were expressed as fold increase compared to Air mice exposed to PBS using *hprt1* expression as a houskeeping gene. IFN-λ2/3 was evaluated by ELISA (pg/mL) **(b)** Histological changes were evaluated at 9 and 12 dpi. Scale bar = 50 µm. Histological score analysis including epithelial damages are expressed as mean ± SEM. WT mice (white bars) and IL-20Rb KO mice (Hashed bars).# $p < 0.05$, ## $p < 0.01$, correspond to IL-20Rb KO effect (IL-20Rb KO vs. WT). Three independent experiments have been performed with 3-5 mice in each group per experiment.

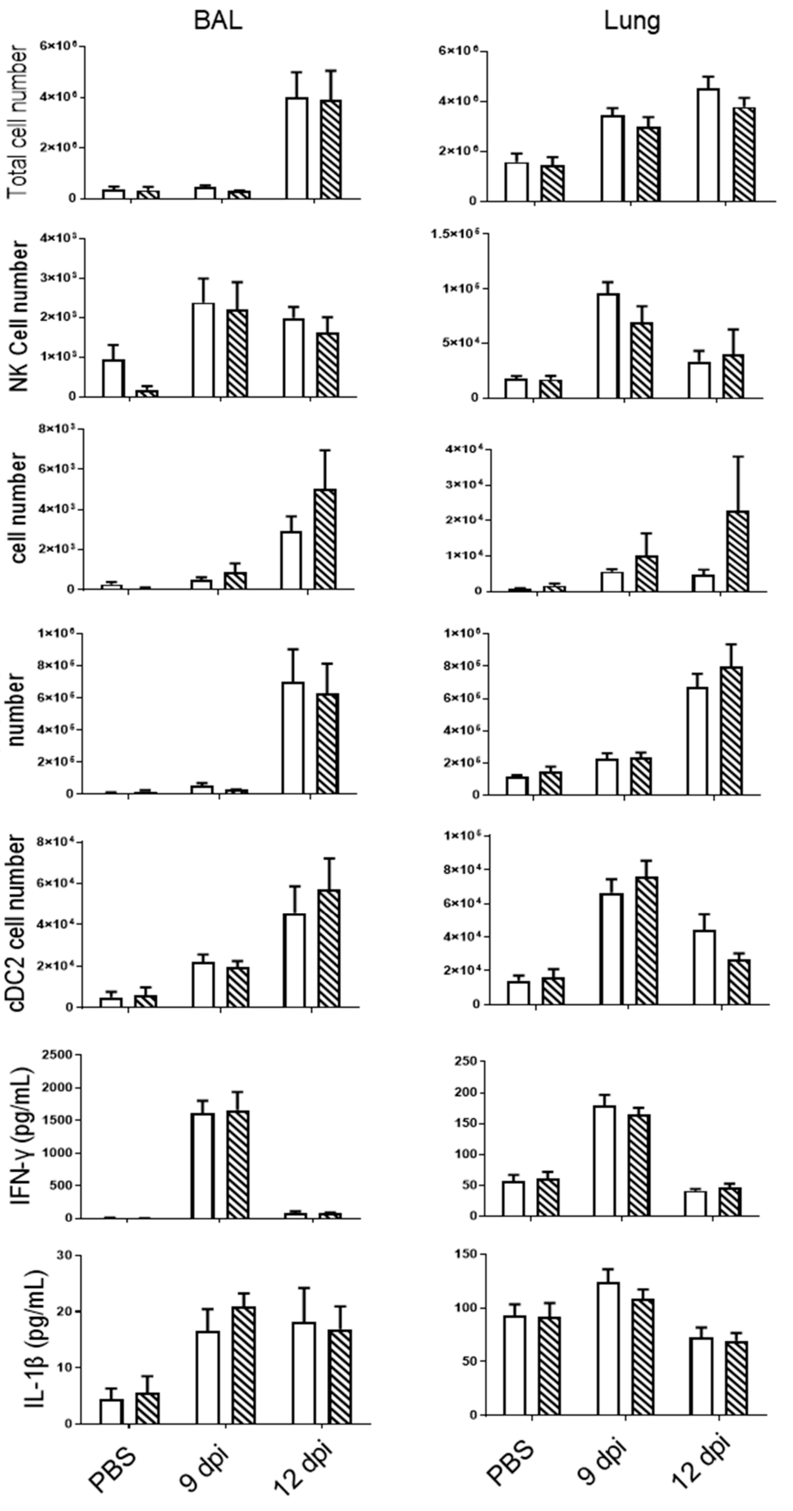

Figure S4. PVM infection induces lung inflammation both in WT and IL-20Rb KO mice. Total cell number, CD45+/TCRβ-/NK1.1+ NK cells, CD45+/TCRβ+/NK1.1+ NKT like cells, CD45+/TCRβ+ CD4+ and CD8+ T lymphocytes and CD45+/ F4/80-/ CD11c+ dendritic cells (cDC2) count were analysed in BAL fluid and lung tissue of WT (white bars) and IL-20Rb KO mice (hashed bars). IFN-γ and IL-1β levels were analysed by ELISA (pg/mL). Data represent the mean ± SEM. Three independent experiments have been performed with 3-5 mice in each group per experiment.

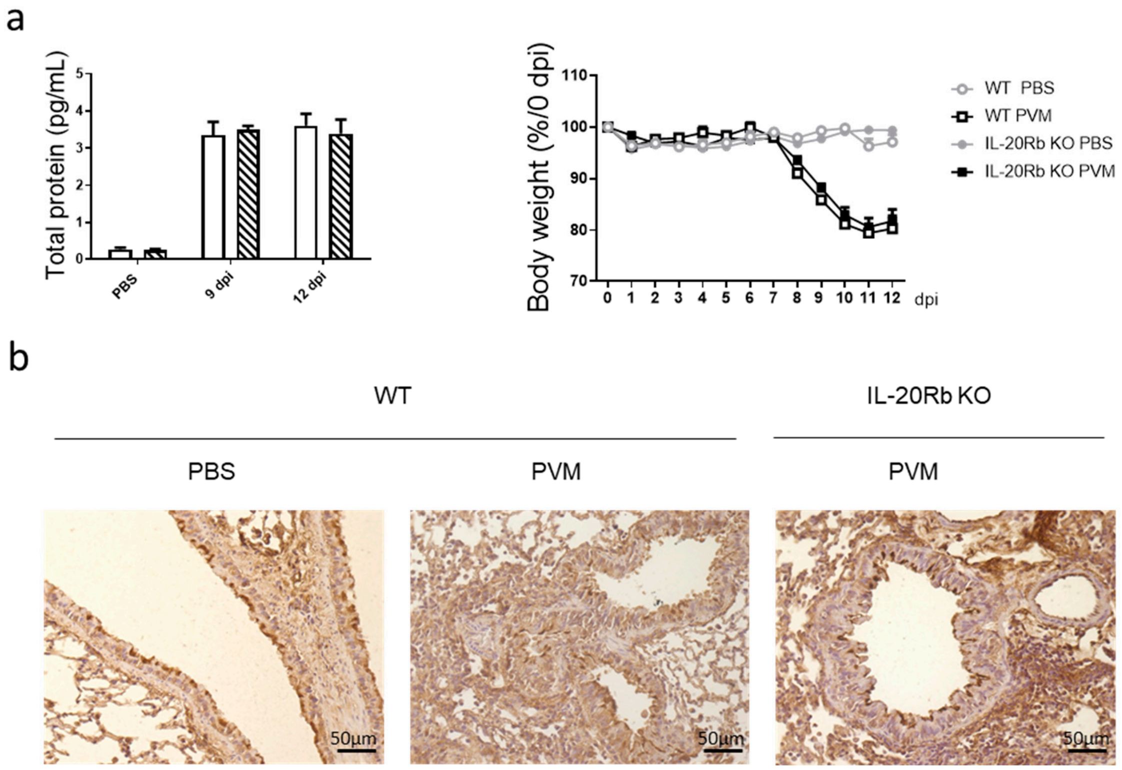

Figure S5. PVM infection affects E-cadherin protein expression in Air mice. (**a**) Lung permeability was analysed through the quantification of total protein in BAL fluid (pg/mL). (White bars) and IL-20Rb KO mice (Hashed bars). The body weight loss was evaluated following PVM infection and was expressed in percentage in comparison to day 0 (**b**) Expression of e-cadherin was evaluated on lung sections by immunohistochemistry at 12 dpi in Air WT mice. Scale bar = 50 µm. Data represent the mean ± SEM. Three independent experiments have been performed with 3-5 mice in each group per experiment..
